# Genotype-specific prevalence of human papillomavirus infection in asymptomatic Peruvian women: a community-based study

**DOI:** 10.1186/s13104-021-05588-7

**Published:** 2021-05-07

**Authors:** Juana del Valle-Mendoza, Lorena Becerra-Goicochea, Miguel Angel Aguilar-Luis, Luis Pinillos-Vilca, Hugo Carrillo-Ng, Wilmer Silva-Caso, Carlos Palomares-Reyes, Andre-Alonso Taco-Masias, Ronald Aquino-Ortega, Carmen Tinco-Valdez, Yordi Tarazona-Castro, Cynthia-Wendy Sarmiento-Ramirez, Luis J. del Valle

**Affiliations:** 1 School of Medicine, Research and Innovation Center of the Faculty of Health Sciences, Universidad Peruana de Ciencias Aplicadas, Lima, Peru; 2 Laboratorio de Biologia Molecular, Instituto de Investigación Nutricional, Lima, Peru; 3 Hospital Regional Docente de Cajamarca, Cajamarca, Peru; 4 Escuela Profesional de Genética y Biotecnología. Facultad de Ciencias Biológicas, Universidad Nacional Mayor de San Marcos, Lima, Peru; 5 Barcelona Research Center for Multiscale Science and Engineering, Departament D’Enginyeria Química, EEBE, Universitat Politècnica de Catalunya (UPC), Barcelona, Spain

**Keywords:** Peru, Human papillomavirus, Cervical cancer, PCR

## Abstract

**Objective:**

To determine the general and genotype-specific prevalence of HPV and to identify potential risk factors for the infection in a population-based screening of Peruvian women.

**Results:**

A total of 524 samples were analyzed by PCR and a total of 100 HPV positive samples were found, of which 89 were high-risk, 19 were probably oncogenic, 9 were low-risk and 27 other HPV types. The 26–35 and 36–45 age groups showed the highest proportion of HPV positive samples with a total of 37% (37/100) and 30% (30/100), respectively. Moreover, high-risk HPV was found in 33.7% of both groups and probably oncogenic HPV in 52.6% and 31.6%, respectively. High-risk HPV were the most frequent types identified in the population studied, being HPV-52, HPV-31 and HPV-16 the most commonly detected with 17.6%, 15.7% y 12.9%, respectively. Demographic characteristics and habits were assessed in the studied population. A total of 62% high-risk HPV were detected in married/cohabiting women. Women with two children showed the highest proportion (33.8%) of high-risk HPV, followed by women with only one child (26.9%). Those women without history of abortion had a higher frequency of high-risk HPV (71.9%), followed by those with one abortion (25.8%).

**Supplementary Information:**

The online version contains supplementary material available at 10.1186/s13104-021-05588-7.

## Introduction

The human papillomavirus (HPV) is a double stranded DNA virus, with worldwide distribution [1, 2]. More than 100 types of human papillomavirus have been identified, including 13 high-risk types, which are responsible for cervical, anogenital and oropharyngeal cancers [2–4]. HPV infection is the most common sexually transmitted infection worldwide, that affects at least 50% of sexually active individuals of both sexes at some point during their life [2, 3]. The prevalence of HPV infection peaks in young sexually active women and decreases after 35 years of age, however, a small second peak occurs in middle-aged women older than 55 years in some developing regions [5, 6].

It has been reported that viral infections contribute to 15–20% of all human cancers, as several viruses facilitate the multistage development of cancers [1]. Most HPV infections are harmless and clear spontaneously within 1–2 years of acquiring it; however, persistent infection with high-risk HPV can lead to cancer [1–3]. HPV 16 and 18 are the most common high-risk types, which are involved in around 70% of all cervical cancer [2]. Cervical cancer is the fourth most common cancer in women in the world, with an incidence rate of 13.1 per 100,000 women/year and nearly half a million of cases yearly [7]. Currently, developing regions such as Latin America have higher rates of cervical cancer, with incidence rates ranging from 10 to 80 per 100,000 women/year [8].

Cervical screening and early treatment have successfully lowered cervical cancer incidence and mortality in developed countries, nonetheless, these measures have not been as effective in Peru [9, 10]. HPV infection and cervical cancer remain a major public health problem in Peru, being the second most common cancer among women and affecting 32 per 100,000 women/year [10, 11].

Since the recent introduction of molecular techniques for detection of HPV, it has become evident that screening with HPV tests protect better against future cancerous lesions than cytology-based screening alone, and consequently, virological screening programs are becoming increasingly recommended [12–14]. It is crucial recognize the epidemiology of HPV in a certain population to provide a better control of the disease, therefore, the objective of this study was to determine the general and genotype-specific prevalence of HPV and to identify potential risk factors for the infection in a population-based screening of Peruvian women.

## Main text

### Methods

#### Patients and study design

A cross sectional study was carried out, using samples and data collected from a previous study performed in *Hospital Regional Docente de Cajamarca*, in northern Peru during September 2017 to July 2019. Asymptomatic women attending a gynecological outpatient health center, who had a history of at least 1 sexual encounter were studied. Exclusion criteria included: pregnancy, severe gynecological bleeding, previous hysterectomy, previous history of HPV-related disease including cancer, warts and cutaneous manifestations.

#### Sample collection

Samples were collected from the ectocervix and endocervix using a disposable cytobrush, they were stored in a tube containing phosphate buffered saline (pH:8.6) for preservation. Three aliquots of each sample were stored at − 20 °C until testing.

#### DNA extraction

Samples were kept at − 20 °C until analyzed. DNA extraction from 200 µL of cervico-vaginal samples was performed using the High Pure PCR Viral Nucleic Acid Kit (Roche Diagnostics GmbH, Mannheim, Germany), according to manufacturer’s instructions.

#### PCR for HPV amplification

HPV amplification was carried out using primers and conditions previously described [15]. The HPV genotypes were classified based on the IARC classification for cancer risk: high, possibly oncogenic and low risk [4].

#### Ethics statement

This study was approved by the Research Ethics Board of the *Hospital Regional de Cajamarca,* Peru. The samples were obtained in the Specialized Oncological Preventive Service of the *Hospital Regional de Cajamarca*, as part of the cervical cancer screening program. All samples were analyzed after a written informed consent was signed.

#### Statistical analysis

All analyses were processed with the IBM Statistical Package for the Social Sciences (SPSS) software version 21.0 (SPSS, Chicago, IL, USA).

Maps were created using QGIS 3.12.3 software. Data for creating the map were acquired from the Instituto Nacional de Estadística e Informática (https://www.inei.gob.pe/).

### Results

A total of 524 samples were analyzed by PCR, which were classified according to its oncogenic potential in 13 high-risk HPV, 12 probably oncogenic HPV and 2 low-risk HPV (Additional file 1: Table S1) The population studied was analyzed according to age groups, the most common group was between 36–45 years old with 35.1% (184/524), followed by 26–35 years old with 31.9% (167/524). A total of 100 patients positive for HPV were found, of which 89 were high-risk, 19 were probably oncogenic, 9 were low-risk and 27 other HPV types. The 26–35 and 36–45 age groups showed the highest proportion of HPV positive samples with a total of 37% (37/100) and 30% (30/100), respectively. Moreover, high-risk HPV was found in 33.7% of both groups and probably oncogenic HPV in 52.6% and 31.6%, respectively (Table 1).

**Table 1:** Demographics in patients Human papillomavirus (HPV) positive

Age range	Totality	HPV positives	Genotype HPV			
	n = 524 (%)	n = 100 (%)	High risk n = 89	Probably oncogenic n = 19	Low risk n = 9	Other HPV types n = 27
≤ 25	48 (9.2)	16 (16.0)	10 (11.2)	1 (5.3)	1 (11.1)	9 (33.3)
26–35	167 (31.9)	37 (37.0)	30 (33.7)	10 (52.6)	6 (66.7)	5 (18.5)
36–45	184 (35.1)	30 (30.0)	30 (33.7)	6 (31.6)	2 (22.2)	8 (29.6)
≥ 46	125 (23.9)	17 (17.0)	19 (21.4)	2 (10.5)	0 (0.0)	5 (18.6)
Total	524 (100.0)	100 (100.0)	89 (100.0)	19 (100.0)	9 (100.0)	27 (100.0)

*HPV genotypes: High risk: 16, 18, 31, 33, 35, 39, 45, 51, 52, 56, 58, 59, 68. Probably oncogenic: 26, 30, 34, 53, 66, 67, 69, 70, 73, 82, 85, 97. Low risk: 6, 11

**Fig. 1:**
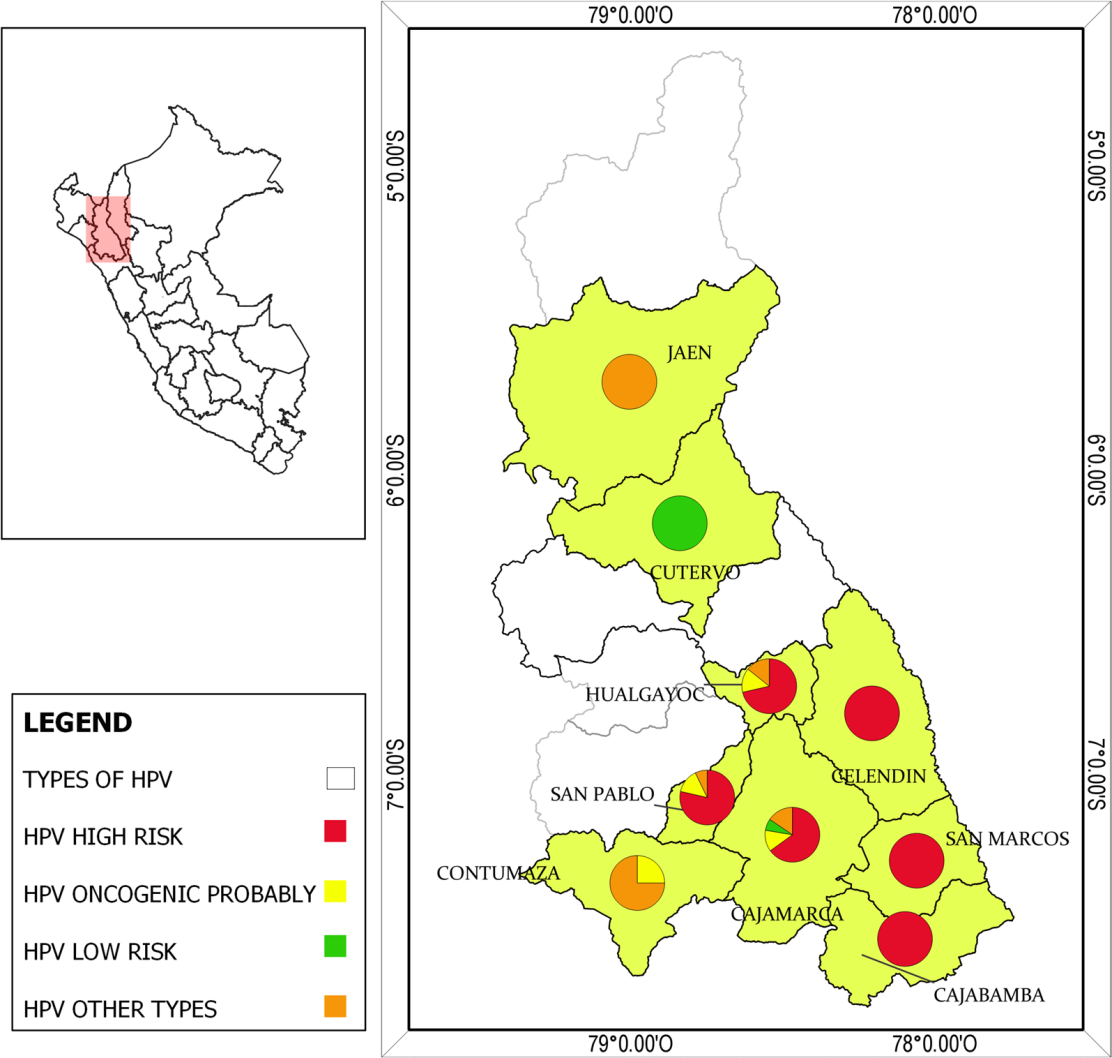
Geographic distribution of infection of HPV in Cajamarca-Peru. Map created by authors using QGIS 3.12.3 software

High-risk HPV were the most frequent types identified in the population studied, being HPV-52, HPV-31 and HPV-16 the most commonly detected with 17.6%, 15.7% and 12.9%, respectively. Followed by types HPV-91 with 14.8%, HPV-71, HPV-43 and HPV-40 with 11.1%, which have not been classified according to its oncogenic potential. Also, probably oncogenic types such as HPV-97, HPV-85, HPV-73 and HPV-34 were not detected in the current study. Finally, HPV-18, another important oncogenic type, was only identified in one sample among all the positive women (Additional file 1: Table S2) (Fig. 1).

Demographic characteristics and habits were assessed in the studied population. A total of 62% high-risk HPV were detected in married/cohabiting women. Women with two or more sexual partners showed a 63% of HPV detection, among these, women who had a sexual partner in the last 6 months showed a frequency of 83% of high-risk HPV. Also, of the total of HPV positive women a 77% did not report condom use, being 75% positive for high-risk HPV (Table 2).

**Table 2:** Demographic characteristics and habits in the studied population

Characteristic	Totality of cases n = 524 (%)	HPV positives n = 100 (%)	HPV types detected			
			High risk n = 89 (%)	Probably oncogenic n = 19 (%)	Low risk n = 9 (%)	Other HPV types n = 27 (%)
Marital status						
Married/cohabiting	388 (74.0)	64 (64.0)	62 (69.7)	15 (79.0)	5 (55.6)	10 (37.0)
Single/separated/divorced/widowed	136 (26.0)	36 (36.0)	27 (30.3)	4 (21.0)	4 (44.4)	17 (63.0)
Number of sexual partners lifetime						
0	0 (0.0)	0 (0.0)	0 (0.0)	0 (0.0)	0 (0.0)	0 (0.0)
1	259 (49.4)	37 (37.0)	37 (41.6)	6 (31.6)	1 (11.1)	7 (26.0)
2	177 (33.8)	40 (40.0)	36 (40.4)	8 (42.1)	2 (22.2)	11 (40.7)
≥ 3	88 (16.8)	23 (23.0)	16 (18.0)	5 (26.3)	6 (66.7)	9 (33.3)
Number of sexual partners in the last 6 months						
0	61 (11.6)	9 (9.0)	6 (6.7)	1 (5.3)	2 (22.2)	6 (22.2)
1	459 (87.6)	91 (91.0)	83 (93.3)	18 (94.7)	7 (77.8)	21 (77.8)
≥ 2	4 (0.8)	0 (0.0)	0 (0.0)	0 (0.0)	0 (0.0)	0 (0.0)
Use of condom						
Yes	109 (20.8)	23 (23.0)	14 (15.7)	5 (26.3)	2 (22.2)	11 (40.7)
No	415 (79.2)	77 (77.0)	75 (84.3)	14 (73.7)	7 (77.8)	16 (59.3)
Use of sex toys						
Yes	2 (0.4)	0 (0.0)	0 (0.0)	0 (0.0)	0 (0.0)	0 (0.0)
No	522 (99.6)	100 (100.0)	89 (100.0)	19 (100.0)	9 (100.0)	27 (100.0)
Extramarital affairs						
Yes	31 (5.9)	8 (8.0)	6 (6.7)	2 (10.5)	0 (0.0)	1 (3.7)
No	493 (94.1)	92 (92.0)	83 (93.3)	17 (89.5)	9 (100.0)	26 (96.3)
Victim of sexual abuse						
Yes	24 (4.6)	6 (6.0)	8 (9.0)	3 (15.78)	0 (0.0)	0 (0.0)
No	500 (95.4)	94 (94.0)	81 (91.0)	16 (84.2)	9 (100.0)	27 (100.0)
Date of the last papanicolaou test						
Never in life	78 (14.9)	11 (11.0)	18 (20.2)	0 (0.00)	2 (22.22)	7 (26.0)
≤ 1 year	238 (45.4)	56 (56.0)	40 (44.9)	5 (26.32)	6 (66.67)	12 (44.4)
≥ 2 years	208 (39.7)	33 (33.0)	31 (34.8)	14 (73.68)	1 (11.11)	8 (29.6)
Number of births						
0	53 (10.11)	17 (17.0)	9 (10.1)	3 (15.79)	5 (55.56)	10 (37.0)
1	107 (20.42)	28 (28.0)	24 (27.0)	3 (15.79)	2 (22.22)	7 (26.0)
2	155 (29.58)	32 (32.0)	30 (33.7)	12 (63.16)	1 (11.11)	5 (18.5)
3	115 (21.95)	13 (13.0)	14 (15.7)	1 (5.26)	0 (0.00)	5 (18.5)
4	51 (9.73)	2 (2.0)	3 (3.3)	0 (0.00)	0 (0.00)	0 (0.0)
≥ 5	43 (8.2)	8 (8.0)	9 (10.1)	0 (0.0)	1 (11.1)	0 (0.0)
Number of abortions						
0	373 (71.2)	73 (73.0)	64 (71.9)	17 (89.5)	9 (100.0)	13 (48.1)
1	121 (23.1)	24 (24.0)	23 (25.8)	0 (0.0)	0 (0.0)	13 (48.1)
2	25 (4.8)	2 (2.0)	0 (0.0)	2 (10.5)	0 (0.0)	1 (3.8)
3	5 (1.0)	1 (1.0)	2 (2.2)	0 (0.0)	0 (0.0)	0 (0.0)

Women with two children showed the highest proportion (33.7%) of high-risk HPV, followed by women with only one child (26.9%). Those women without history of abortion had a higher frequency of high-risk HPV (71.9%), followed by those with one abortion (25.8%). History of sexual abuse and extramarital affairs did not show a high frequency of HPV detection. Finally, 56% of women that underwent Pap test within one year or less were positive for HPV, with 44.9% presenting high-risk types (Table 2).

### Discussion

The understanding that persistent infection with high-risk HPV is crucial for the pathogenesis of cervical cancer has led to new approaches for primary and secondary prevention [12]. Vaccines targeting HPV16 and HPV18, the two most common oncogenic types, were introduced years ago; however, despite vaccination, early screening remains the most significant method to decrease deaths attributable to cervical cancer [12, 16]. Developed countries have shown that cervical cancer rates can decrease with cytology-based primary screening; however, these programs have been difficult to implement in resource-limited countries [17]. HPV testing has a high sensitivity and negative predictive value; therefore, it identifies women at higher risk of cervical cancer, which can be treated with a more specific protocol [16, 17]. Moreover, HPV testing can be used for epidemiological studies, as HPV prevalence from a specific region measures the general risk for developing cervical cancer in that territory [18, 19].

In the present study, we determined the general and genotype-specific prevalence of HPV in a population-based screening of asymptomatic women in Cajamarca, Peru. This epidemiological information and potential predictors of infection are crucial to evaluate vaccination and achieve cervical cancer control in the region.

Firstly, we could highlight the significant prevalence (19.1%) found in the study, which is higher than global pooled prevalence of 11% according to two meta-analyses [19, 20]. Moreover, the prevalence in this study peaks in young women of reproductive age, which is similar to previous literature [5, 6, 20]. For example, Steben et al. [21] reported that detection of HPV was higher among women aged 20–24 years old with a 44.8% of the total cases. Although, it has been recognized that regions like Latin America show a second peak in middle-aged women of around 55 years old [6, 20], the present study showed a decline in the prevalence of women aged 46 or older.

Regarding specific genotypes, this study found that types 52, 16 and 31 were the most frequent high-risk HPV, with 17.6%, 15.7% and 12.9%, respectively. Conversely, type 18 was only found in one sample. According to literature, HPV-16 and HPV-18 are associated with approximately 70% of all cervical cancers [2], moreover, both genotypes have been the most commonly detected in women with normal cytology [6, 20]. A systematic review revealed that the most common HPV types identified were HPV-16 (9.5%), HPV-18 (6.2%) and HPV-33,52 and 58 (0.3%) [22]. The findings in the present study are not consistent with previous estimates, although HPV prevalence is highly variable according to the territory, we should emphasize the high prevalence of HPV-52 and 31 among the samples studied.

HPV infection was also evaluated according to some characteristics of the population to identify potential predictors for infection. We could highlight the greater prevalence among married and cohabiting women, which differs from a study carried out by Castellague [23] that found a higher occurrence of HPV in divorced women. Regarding the number of sexual partners, previous literature evidenced that women with 5 or more sexual partners had the highest proportion of cases with 67%, followed by women with 2–4 sexual partners [23, 24]. This differs from our findings in which women with 1 and 2 sexual partners had higher prevalence than those with 3 or more partners, moreover most of these infections were with high-risk types.

Sexual behavior and habits are important determinants for HPV infection, as infection is most commonly acquired sexually. One of the most important factors that may have an effect on HPV transmission is condom use. A study carried out by Winer et al. [25] showed a low probability of infection with high-risk genotypes among women whose partner consistently used condom in the last eight months. Improper use of condom has also been associated with a higher risk for acquiring HPV infection, nearly doubling the risk of infection compared to correct use [18]. Our data show that women whose partners did not use condom had a higher detection of HPV and high-risk genotypes (89%). Data regarding condom use and its protective role on HPV infection remains inconsistent, however appropriate use of this tool may reduce transmission [25–27].

Another habit evaluated was the use of sexual toys, a previous study on university women showed that these devices are used in approximately 65% of sexual encounters [28], moreover HPV was identified in 75% of women who reported use of vibrators. In the present study, only a 2% of the total population reported use of sexual toys and no cases of infection was detected. Finally, in this study less than 50% of women enrolled did not undergo a Papanicolau (Pap) smear during the last year, being this group of women the one with the highest prevalence of high-risk HPV. This finding differs from the current guidelines by different international and national organizations that recommend screening with Pap smear and/or HPV testing depending on the age and risk factors [29–32].

In conclusion a high prevalence was found among Peruvian women, being the ones between 26–35 years-old and those married/cohabiting the most commonly affected. Also, it was observed a high frequency of high-risk HPV, being types 52, 16 and 31 the most commonly identified. This study highlights the importance to detect the general and genotype-specific prevalence in different populations, as these are important epidemiological tools. Screening with cytological and molecular methods are recommended particularly in women with risk factors.

## Limitations

Firstly, an important limitation is that other sexually transmitted diseases were not evaluated. Also, general and specific genotype distribution of HPV varies greatly among geographic areas, therefore, the current information cannot be extrapolated to substantial different settings.

## Supplementary Information

**Additional file 1: Table S1.** Human papillomavirus types and oncogenic potential. **Table S2.** Most frequent HPV types identified in the population studied.

## Data Availability

Abstraction format used in the study and dataset are available and accessible from the corresponding author upon request in the link: https://figshare.com/s/f286fcb67120a72a7a07.

## Abbreviations

HPV: Human papillomavirus
Pap: Papanicolau
PCR: Polymerase chain reaction
DNA: Deoxyribonucleic acid
bp: Base pairs
