# Supplementary material for: Genotype-specific prevalence of human papillomavirus infection in asymptomatic Peruvian women: a community-based study
Source: BMC Res Notes. 2021 May 7;14:172. doi: 10.1186/s13104-021-05588-7 (PMC8103758; doi:10.1186/s13104-021-05588-7)
Supplement: Supplementary file 1 — Additional file 1: Table S1. Human papillomavirus types and oncogenic potential. Table S2. Most frequent HPV types identified in the population studied. [file 13104_2021_5588_MOESM1_ESM.docx]

**Table S1. Human papillomavirus types and oncogenic potential**

| **Classification** | **HPV types** |
| --- | --- |
| **High risk (group 1/2A)^a^** | 16, 18, 31, 33, 35, 39, 45, 51, 52, 56, 58, 59, 68 |
| **Probably oncogenic (group 2B)^b^** | 26, 30, 34, 53, 66, 67, 69, 70, 73, 82, 85, 97 |
| **Low risk** | 6, 11 |

Adapted from Bouvard et al. [[4]](https://www.sciencedirect.com/science/article/pii/S1569905616300999#bib0010).

HPV = human papillomavirus.

a. Sufficient evidence for cervical cancer

b. Limited evidence for cervical cancer and classified by phylogenetic analogy to HPV types 1/2A

**Table S2: Most frequent HPV types identified in the population studied.**

| **Genotypes detected High risk (included probably oncogenic)** | **Frequency n = 108** | **Percentage (%)** |
| --- | --- | --- |
| HPV16 | 17 | 15.7 |
| HPV18 | 1 | 0.9 |
| HPV31 | 14 | 12.9 |
| HPV33 | 8 | 7.4 |
| HPV35 | 3 | 2.8 |
| HPV39 | 4 | 3.7 |
| HPV45 | 2 | 1.9 |
| HPV51 | 7 | 6.5 |
| HPV52 | 19 | 17.6 |
| HPV56 | 4 | 3.7 |
| HPV58 | 3 | 2.8 |
| HPV59 | 1 | 0.9 |
| HPV68 | 6 | 5.6 |
| HPV26 | 2 | 1.9 |
| HPV30 | 2 | 1.9 |
| HPV53 | 4 | 3.7 |
| HPV66 | 3 | 2.8 |
| HPV67 | 4 | 3.7 |
| HPV69 | 2 | 1.6 |
| HPV70 | 1 | 0.9 |
| HPV82 | 1 | 0.9 |
| **Low risk** | **n = 9** |  |
| HPV6 | 5 | 55.6 |
| HPV11 | 4 | 44.4 |
| **Other HPV types** | **n = 27** |  |
| HPV14 | 1 | 3.7 |
| HPV20 | 1 | 3.7 |
| HPV21 | 1 | 3.7 |
| HPV40 | 3 | 11.1 |
| HPV43 | 3 | 11.1 |
| HPV44 | 2 | 7.4 |
| HPV54 | 1 | 3.7 |
| HPV55 | 2 | 7.4 |
| HPV71 | 3 | 11.1 |
| HPV74 | 2 | 7.4 |
| HPV80 | 1 | 3.7 |
| HPV81 | 1 | 3.7 |
| HPV90 | 1 | 3.7 |
| HPV91 | 4 | 14.8 |
| HPV101 | 1 | 3.7 |
